# Caution Is Warranted When Assessing Diastolic Function Using Transesophageal Echocardiography. Comment on Kyle et al. Consensus Defined Diastolic Dysfunction and Cardiac Postoperative Morbidity Score: A Prospective Observational Study. *J. Clin. Med.* 2021, *10*, 5198

**DOI:** 10.3390/jcm11113105

**Published:** 2022-05-31

**Authors:** Filippo Sanfilippo, Luigi La Via, Simone Messina, Bruno Lanzafame, Veronica Dezio, Marinella Astuto

**Affiliations:** 1 Department of Anesthesiology and Intensive Care, AOU “Policlinico—San Marco” Catania, 95123 Catania, Italy; luigilavia7@gmail.com (L.L.V.); veronicadezio8@gmail.com (V.D.); astmar@tiscali.it (M.A.); 2 School of Specialization in Anesthesiology and Intensive Care, University “Magna Graecia”, 88100 Catanzaro, Italy; messina.simone05@gmail.com; 3 Department of Anesthesiology and Intensive Care, AO “Umberto I”, ASP Siracusa, 96100 Siracusa, Italy; lanza.bb@gmail.com

Kyle et al. [1] conducted a very interesting study evaluating the impact of left ventricular diastolic dysfunction (LVDD) in patients undergoing cardiac surgery, confirming that presence of any grade LVDD is associated with greater burden of morbidity. These results are not surprising since the presence of LVDD has been linked to worse patient’s outcomes also outside from cardiac surgery, both in hospitalized adult patients and in the pediatric setting [2,3,4,5,6]. The authors should be congratulated for this study, but we have some remarks to make. 

First of all, the authors applied the latest guidelines for the diagnosis and grading of LVDD [7], but when relying on a transesophageal assessment, the evaluation of the left atrial (LA) volume becomes unreliable. Indeed, in the mid-esophageal region the transesophageal echocardiography (TEE) probe sits just behind the LA, making it impossible to image the full atrium. Therefore, we would suggest caution in slavishly applying the latest guidelines when using TEE, and the authors may wish to clarify how they measured LA with TEE. Moreover, the latest guidelines rely on tissue Doppler assessment (e’ and E/e’ ratio) [7]; however, their use in TEE may not be straightforward. Indeed, Mauermann et al. recently showed that e’ measurements performed in the mid-esophageal four-chambers view are significantly underestimated as compared to the transthoracic echocardiography apical four-chambers view, with a mean within-patient difference of 0.6 cm/s [8]. Consequently, the E/e’ ratio is overestimated by TEE. 

In general, the use of TEE is a well-established cornerstone in cardiac surgery [9,10,11,12], and it is very useful also outside of the cardiac surgery setting [13]. It must be emphasized that TEE has several merits in the setting of patients undergoing cardiac surgery, allowing for instance the evaluation of left ventricular systolic function and of right ventricular function, and also providing useful information for fluid management [14]. However, the application of the latest guidelines for LVDD assessment with TTE should be performed with caution and keeping in mind the above-described limitations [15].

Finally, we think that the study may benefit from full adherence to the checklist for reporting echocardiography research studies suggested by the recently published recommendations known as “Preferred Reporting Items for Critical-care Echocardiography Studies (PRICES)” [16,17]. The PRICES expert consensus aims at providing guidance on reporting echocardiography research; full adherence with these recommendations may improve not only the interpretation of the study in itself, but may also render easier between-study comparison with new research enhancing this study with external validation.
